# Immediate health and wellbeing benefits of short-term forest therapy for urban healthcare workers: a case study in Giant Panda National Park with cultural ecosystem services

**DOI:** 10.3389/fpsyg.2025.1630999

**Published:** 2025-11-18

**Authors:** Ping Zhang, Yixin Cui, Rui Liu, Yifei Zhao, Wenjun Su, Li Zhao, Xiaohua Wang, Li Deng, Boya Wang, Jinpeng Li, Yanbin Yang, Mingze Chen, Weiquan Guo, Lilin Song, Qingjie Zhang, Fuxi Xie, Saixin Cao, Guangyu Wang, Tongyao Zhang, Shihong Yang, Xi Li

**Affiliations:** 1 College of Landscape Architecture, Sichuan Agricultural University, Chengdu, China; 2 School of Architecture and Urban Planning, Shenzhen University, Shenzhen, China; 3 Tangjiahe Area of the Giant Panda National Park, Guangyuan, China; 4 Sichuan Forestry Central Hospital, Chengdu, China; 5 Department of Landscape Architecture and Urban Planning, College of Architecture, Texas A&M University, College Station, TX, United States; 6 School of Mechanical Engineering, University of Science and Technology Beijing, Beijing, China; 7 Department of Forest and Resources Management, Faculty of Forestry, University of British Columbia, Vancouver, BC, Canada; 8 Whenchuan Hospital of Traditional Chinese Medicine, Wenchuan, China; 9 Forestry and Grassland Administration of Sichuan Province, Chengdu, China

**Keywords:** Giant Panda National Park, forest therapy, CES, health and wellbeing, urban healthcare workers

## Abstract

The increasing frequency and prominence of global public health threats put urban healthcare workers at risk of physical and mental illness. Forest therapy holds crucial importance in promoting human health as a non-material benefit obtained from ecosystems. Cultural ecosystem services (CES) profoundly influence human welfare. National parks, due to their rich biodiversity and other favorable conditions, can support forest therapy and provide CES. This study organized 32 urban healthcare workers to participate in a two-day, two-night forest therapy in the Giant Panda National Park (GPNP), which provides CES, and examined immediate changes in their physical and mental health before and after the intervention. Among these, physiological indicators encompass respiratory and circulatory system, immune system, neurotransmitter system, physical fitness development, and sleep quality. Psychological indicators include self-restore and preferences, sensory perception, transcendent experiences, and personal subjective wellbeing. The results indicate that both forest bathing and sensory therapy activities within the GPNP may yield varying degrees of relaxation and concentration benefits. Forest therapy in medium hydrodynamic landscapes may offer significant physiological relaxation benefits for high-stress groups such as urban healthcare workers, while sensory therapy in forest environment may positively enhance concentration levels. Activities such as observation and experiential learning within national parks characterized by pristine ecological environments may be more effective in evoking positive or even exhilarating emotions. This exploratory finding could potentially contribute to the rehabilitation treatment of individuals with depression. Research findings on respiratory and circulatory systems, immune systems, neurotransmitter systems remind us that culture and nature are not in conflict. Infusing cultural elements into sufficiently good ecological environments may bring greater benefits to humanity. This exploratory discovery could aid future selections of therapeutic microenvironments for sub-health populations and individuals with respiratory diseases. This study also found that the most contributing activities to the mental health of urban healthcare workers in different environments were not exactly the same, with Baduanjin, plant nameplates and mandalas, and meditation on positive thoughts being highly contributing to both types of environments, while the landscape of smell was more contributing in the waterside environment of a national park, and the activity of embracing trees was more contributing in the forested environment of a national park. Additionally, the mental health benefits derived from natural environments with cultural ambiance surpass those of forest bathing in purely natural settings, which aligns with our findings regarding physiological benefits. The exploratory findings of this study may provide scientific evidence for the comprehensive impact of national parks on human health, and to offer feasible nature-based solutions for the health and wellbeing of urban healthcare workers and the broader population.

## Highlights

- Sensory Therapy F in Giant Panda National Park yielded physiological health effects of higher concentration compared with Sensory Therapy W and Forest Bathing.
- The CES values obtained by healthcare workers in Giant Panda National Park environment and the implementation of forest bathing and sensory health activities in different environments can produce psychological health and personal wellbeing effects.
- The perceived values of aesthetics, biodiversity, sustaining life, spirituality, recreation, sense of place, and healing were higher.
- The most contributing activities to the mental health of healthcare workers in different environments were not exactly the same.
- Baduanjin, botanical nameplates and mandalas, and meditating on positive thoughts being highly contributing to both types of environments, while the activity of aroma-viewing recreation was more contributing in the waterside environment of a national park, and the activity of embracing a large tree was more contributing in the forested environment of a national park.

## 1 Introduction

In recent years, to address global public health emergencies, the sustained overload of medical work has significantly increased the prevalence of related illnesses among urban healthcare workers (Sugawara et al., 2017). Surveys reveal that the morbidity rate among urban healthcare workers has reached a staggering 52.8%, including musculoskeletal disorders, cardiovascular diseases, as well as neurological and psychological conditions (Zhong et al., 2021; Song et al., 2020). Among these, the most common psychological issues include anxiety (26.5%−44.6%), depression (8.1%−25.0%), and insomnia (23.6%−38.0%) (García-Iglesias et al., 2020). Prolonged anxiety severely impacts the physical health and work efficiency of urban healthcare workers (Bargellini et al., 2000). Therefore, focusing on the health status of urban healthcare workers is a critical societal issue closely tied to public wellbeing.

Nature holds significant value for human development. Forests, with their clean air and comfortable microclimates, have become ideal sites for therapeutic and health-promoting activities (Deng, 2016). Health is defined as a holistic state of physical, psychological, and social wellbeing (World Health Organization, 2022). According to the Attention Restoration Theory (ART) and the Stress Reduction Theory (SRT), the natural environment contains numerous therapeutic factors that deliver significant health benefits through sensory input such as sight, smell, sound, or touch. People can not only recover from depleted attentional resources in natural environments (Kaplan, 1995; Ulrich, 1983; Ulrich et al., 1991), but also gain constructive benefits, such as enhanced physical activity and transcendental experiences, including humility, awe (Ballew and Omoto, 2018), and introspection (Kaplan and Kaplan, 1989). Concepts such as forest bathing and forest therapy have been proposed in academia, focusing on health activities conducted in forest environments to achieve physical and mental relaxation. This form of intervention aims to utilize nature's psychological and physiological benefits for medical prevention rather than drug treatment (Rosa et al., 2021).

Recent studies have increasingly emphasized the importance of forest environments as health-promoting settings, highlighting the therapeutic potential of forest therapy. Research indicates that forest therapy has restorative effects on the human body (Liu et al., 2018; Shao and Wang, 2020), and can alleviate stress states and induce physiological relaxation (Lee J. et al., 2014; Song et al., 2017). Yu et al. (2017) conducted an experiment demonstrating the positive effects of short-term forest therapy on the heart rate and blood pressure of middle-aged and elderly individuals. Forest walking has been shown to lower stress hormone levels, relax the mind, and improve sleep quality (Lin et al., 2022). Ideno et al. (2017) suggest that the autonomic nervous system plays a crucial role in blood pressure regulation, and that norepinephrine levels and heart rate changes can reflect autonomic nervous system activity. Furthermore, studies have indicated that forest walking can improve immune function (Le Gear et al., 2023). After spending time in forest settings, the expression of perforin by natural killer cells and CD8+ T-lymphocytes is significantly decreased, and the production of pro-inflammatory cytokines, including interferon-gamma and interleukin-6 has a significant reduction. Deng et al. (2023) reported that short-term interventions in forest settings can significantly improve pulmonary ventilation function in patients with COPD, particularly in women compared to men. From the psychological aspect, forest therapy positively impacts stress recovery, nature connectedness, and creativity (Keller et al., 2024; Subirana-Malaret et al., 2023). Relevant research emphasizes that connecting with nature can improve wellbeing, promote cognitive restoration, and reduce stress (Vella-Brodrick and Gilowska, 2022). Yu et al. (2022) point out that volatile organic compounds released by trees, such as phytoncides, can alleviate negative emotions and induce positive moods. Other studies have validated the efficacy of forest therapy in enhancing sleep and reducing emotional stress among medical workers (Kim et al., 2022), and Kavanaugh et al. (2022) propose that healthcare providers engage in regular forest bathing practices to alleviate stress and professional burnout. The findings from scholars suggest that forest therapy can provide a valuable method for psychological relaxation.While a substantial body of research has explored the physiological and psychological benefits of forest therapy, the focus has predominantly been on specific urban environments and suburban forests (Yu and Hsieh, 2020; Ramanpong et al., 2025). However, research on forest therapy in protected areas, such as National Parks and Biosphere Reserves, remains relatively scarce. This leaves a gap in our understanding of the potential benefits of forest therapy in these regions. Giant Panda National Park (GPNP), one of China's earliest national parks, features biodiverse and visually captivating forest environments. It provides unique opportunities for interaction with nature, such as walking and meditating in ancient forests, which merit further investigation into their physiological and psychological health benefits.

Cultural ecosystem services (CES) are defined as “the non-material benefits people derive from ecosystems through spiritual enrichment, cognitive development, recreation, and aesthetic experiences” (World Resources Institute, 2005). According to Díaz et al. (2006), human wellbeing is “a human experience encompassing the basic materials for a good life, freedom and choices of action, health, good social relationships, cultural identity, and a sense of security.” Research on CES in protected areas often employs interdisciplinary methods to assess, quantify, and map CES experiences of local residents and visitors (Ament et al., 2017; Crouzat et al., 2022; Gan et al., 2024; Felipe-Lucia et al., 2024), providing insights into human-nature interactions. CES encompass multiple dimensions of human wellbeing, including mental and physical health, identity, a sense of belonging, and inspiration (Russell et al., 2013; Bryce et al., 2016). To date, several studies have explored the benefits of perceived CES for human wellbeing. Cabana et al. (2024) explored the linkages between CES in Sardinia's coastal environments and the subjective wellbeing of adolescents, highlighting the importance of specific landscape features (e.g., coasts, beaches, dunes, and pine forests) and abiotic factors in promoting positive emotions. Xia et al. (2024) reported on residents' perceptions of the importance of CES and their association with subjective wellbeing in a peri-urban area of Shanghai. However, research remains insufficient regarding the associations between CES in broader and even specific spaces (such as World Heritage Sites, internationally important wetlands, global geoparks, and protected areas like national parks) and the health and wellbeing of broader populations, including vulnerable groups. To bridge this gap, it is essential to explore the potential roles CES play in enhancing human wellbeing, particularly their connections to health and wellbeing across broader populations, including vulnerable groups.

Traditionally, vulnerable groups are defined as “socially marginalized populations, often due to low socioeconomic status, low income, lack of access to basic services such as healthcare, lack of power, gender, or limited access to communication technologies” (Pachauri and Mayer, 2015). However, urban high-stress, middle-to-high-income groups, such as urban healthcare workers, face significant health pressures due to occupational characteristics and societal factors. These groups should also be considered potential vulnerable populations. Therefore, dedicated research is required to comprehensively understand the impact of CES on their health and wellbeing.

Based on the literature documented above, we predict that short-term forest therapy in GPNP will support the health of urban healthcare workers. Following the interventions, immediate physiological improvements in heart rate, blood pressure, immunity indicators, and pulmonary ventilation function are expected; immediate psychological improvements in wellbeing, restoration, and sleep quality are expected. Meanwhile, we further hypothesize that CES (as a secondary contextual element) provides support to urban healthcare workers within the GPNP. Psychologically, immediate improvements in restorative experience, positive emotions, stress relief, and physiological-cognitive responses are anticipated. Therefore, in this study, we focus on investigating the immediate health and wellbeing benefits of forest therapy and CES in GPNP for urban healthcare workers. The study seeks to address the following questions:

1. Do forest therapy (as the primary research focus) and CES (as a secondary contextual element) in GPNP have identifiable immediate physiological and psychological health benefits for urban healthcare workers? If positive physiological and psychological health benefits indeed exist, what evidence supports these effects?
2. What and how specific forest environments, activities, and CES attributes contribute to improvements in immediate psychological health and overall wellbeing?

## 2 Methods

### 2.1 Research participants

Starting from June 2024, we recruited 32 (based on the requirements of previous research experience and literature review, the sample size for similar experiments should generally be no fewer than 30 participants) urban healthcare workers (14 males, 18 females, all of them signed an information letter) in Chengdu, Sichuan, for the experiment. To minimize the impact of differences in sociological demographics on the experimental results, we considered the stability and comprehensiveness of participants' socio-demographic characteristics when recruiting volunteers. The age range of the participants was 24–55 years, and the grouping was done in such a way that the number of people in the same age range in each group remained convergent (i.e., a total of eight people in each group, two people in the age range of 24–34 years, three people in the age range of 35–44 years, and three people in the age range of 45–55 years), and after this condition was met, the people were randomly assigned. All of the above employ the lottery method within simple randomization to randomly assign participants to different groups for observation. All participants were clinical doctors, including those from pediatrics, obstetrics and gynecology, oncology, surgery, and internal medicine, and all groups of participants included all departments and converged in number (i.e., one in pediatrics, one in obstetrics and gynecology, two in oncology, two in surgery, and two in internal medicine in each group). Their average monthly income was RMB 7,000–9,000 (Table 1). Their daily diets were generally light, and they had no history of drug abuse, alcoholism, or smoking, nor did they experience motion sickness. During the week before the experiment, the 32 participants worked at a comparable intensity, all worked a 1-day night shift, and did not work a night shift or undergo a major surgery on the day before the experiment. All participants abstained from consuming beverages or medications that could affect mood, mental state, or sleep for 3 days prior to the experiment. Female subjects were all in a non-menstrual phase. At the same time, interview data indicated that the participants frequently felt fatigued due to work-related stress, had a strong desire to visit green spaces, but their low frequency of visits was attributed to the high workload and time constraints. Informed consent was obtained from all participants, which included consent for human images. This study adheres to the Declaration of Helsinki and was approved by the Ethics Committee of Sichuan Forestry Center Hospital.

**Table 1:** Demographic characteristics of the participants.

**Demographics**	**Composition**	**Number**	**(%)**
Gender	Male	14	43.8
	Female	18	56.2
Age (years old)	24–34	8	25.0
	35–44	12	37.5
	45–55	12	37.5
Department	Pediatrics	4	12.5
	Obstetrics and gynecology	4	12.5
	Oncology	8	25.0
	Surgery	8	25.0
	Internal medicine	8	25.0
Income (RMB)	7,000–9,000	32	100
Total		32	100

### 2.2 Experimental area and design

**Figure 1:**
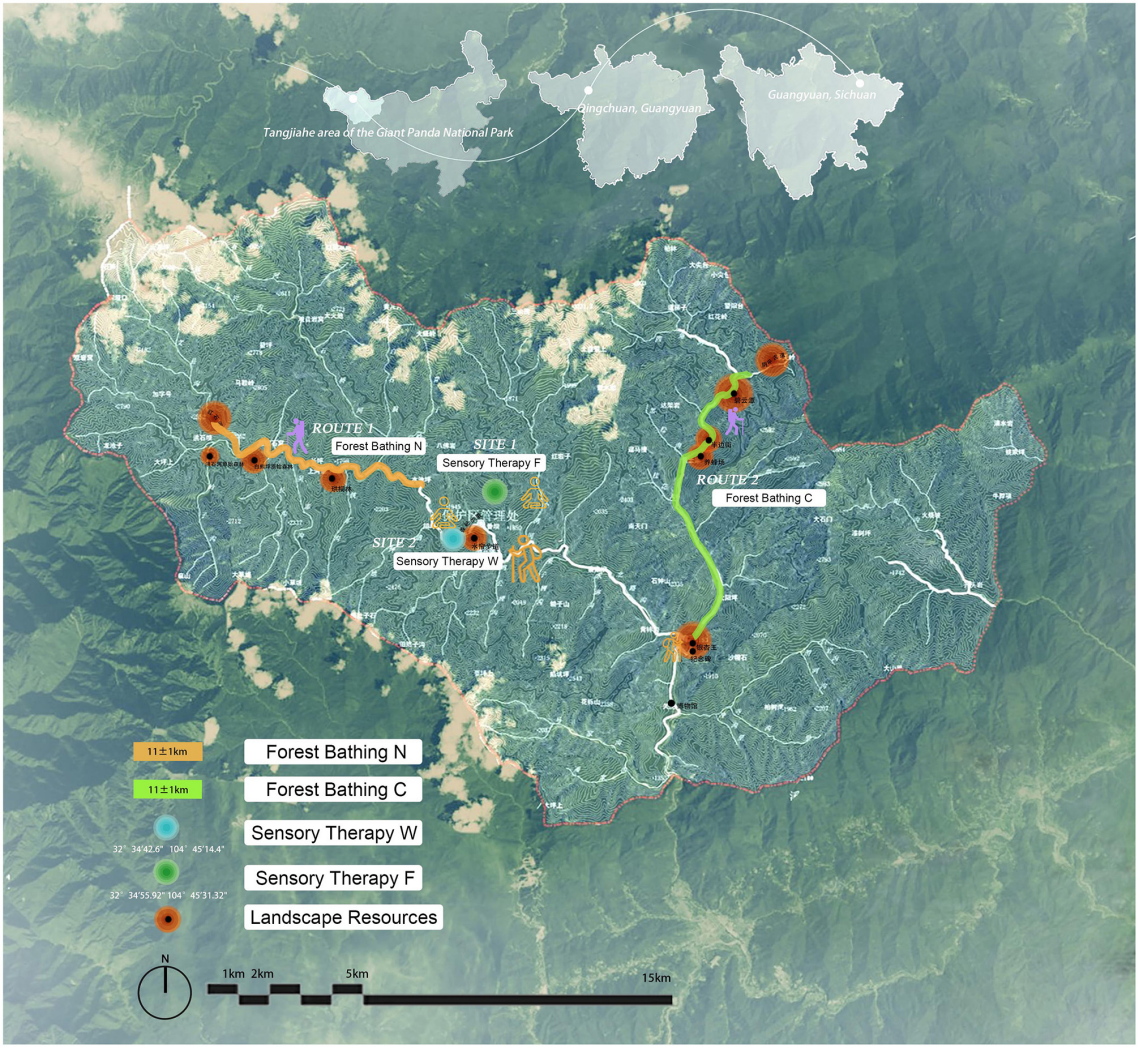
Experimental area and route.

Based on preliminary research, the study selected the Tangjiahe area of the GPNP as the experimental site, located in Qingchuan County, Guangyuan City, Sichuan Province. The climate here is a northern subtropical humid monsoon climate, characterized by four distinct seasons. This area has a well-preserved natural ecosystem and abundant biological resources, making it one of the world's most biodiversity-rich hotspots and the region with the highest encounter rates of rare wildlife in low-altitude areas globally. Based on preliminary surveys and the requirements of this study, the linear and point spaces for conducting this experimental activity were determine (Figure 1). During the experimental period, staff meticulously recorded meteorological and environmental quality data of the experimental area, including representative core health factors of forest therapy: negative Oxygen Ions (NOI). Human comfort factors included Ambient Temperature (AT), Ambient Humidity (AH), Atmospheric Pressure (AP), Total Radiation Instantaneous (TRI), Wind Direction (WD), Instantaneous Wind Speed (IWS), and Sound Pressure Level (SPL). The representative environmental cleanliness factor was PM2.5 (Tables 2, 3).

**Table 2:** Environmental Data for FBN, STW, and STF.

**Time**	**Core health factors of forest therapy**	**Human comfort factors**						**Environmental cleanliness factor**
	**NOI (per/cm**3**)**	**AT (**°**C)**	**AH (%RH)**	**AP (hPa)**	**TRI (W/m** ^2^ **)**	**WD (**°**)**	**IWS (m/s)**	**PM2.5 (**μ**g/m**3**)**
6.11	5672	21	63.68	876.98	268.33	121.42	0.89	7.90
6.12	6415	19	68.57	878.60	134.00	110.04	0.26	7.21
6.13	5532	21	67.63	878.24	240.75	137.23	0.31	6.67
6.14	5760	21	70.54	879.22	192.96	132.77	0.25	7.35
6.15	6696	17	71.70	878.95	98.52	119.40	0.29	7.56

**Table 3:** Environmental data for FBC.

**Time**	**Core health factors of forest therapy**	**Human comfort factors**						**Environmental cleanliness factor**
	**NOI (per/cm**3**)**	**AT (**°**C)**	**AH (%RH)**	**AP (hPa)**	**TRI (W/m** ^2^ **)**	**WD (**°**)**	**IWS (m/s)**	**PM2.5 (**μ**g/m**3**)**
6.11	5478	19	68.40	877.40	116.00	233.00	0.50	7.00
6.12	6594	17	63.76	868.62	88.19	225.62	0.22	5.86
6.13	5970	18	61.48	858.21	120.49	211.41	0.62	4.05
6.14	6446	20	67.32	879.00	88.78	242.22	0.45	8.97
6.15	6487	20	68.08	867.96	102.92	182.92	0.33	7.00

In this study, forest therapy includes forest bathing (experimental site as linear space) and sensory therapy activities (experimental site as point space), as detailed below:

#### 2.2.1 Walking and breathing exercise experimental site

**Figure 2:**
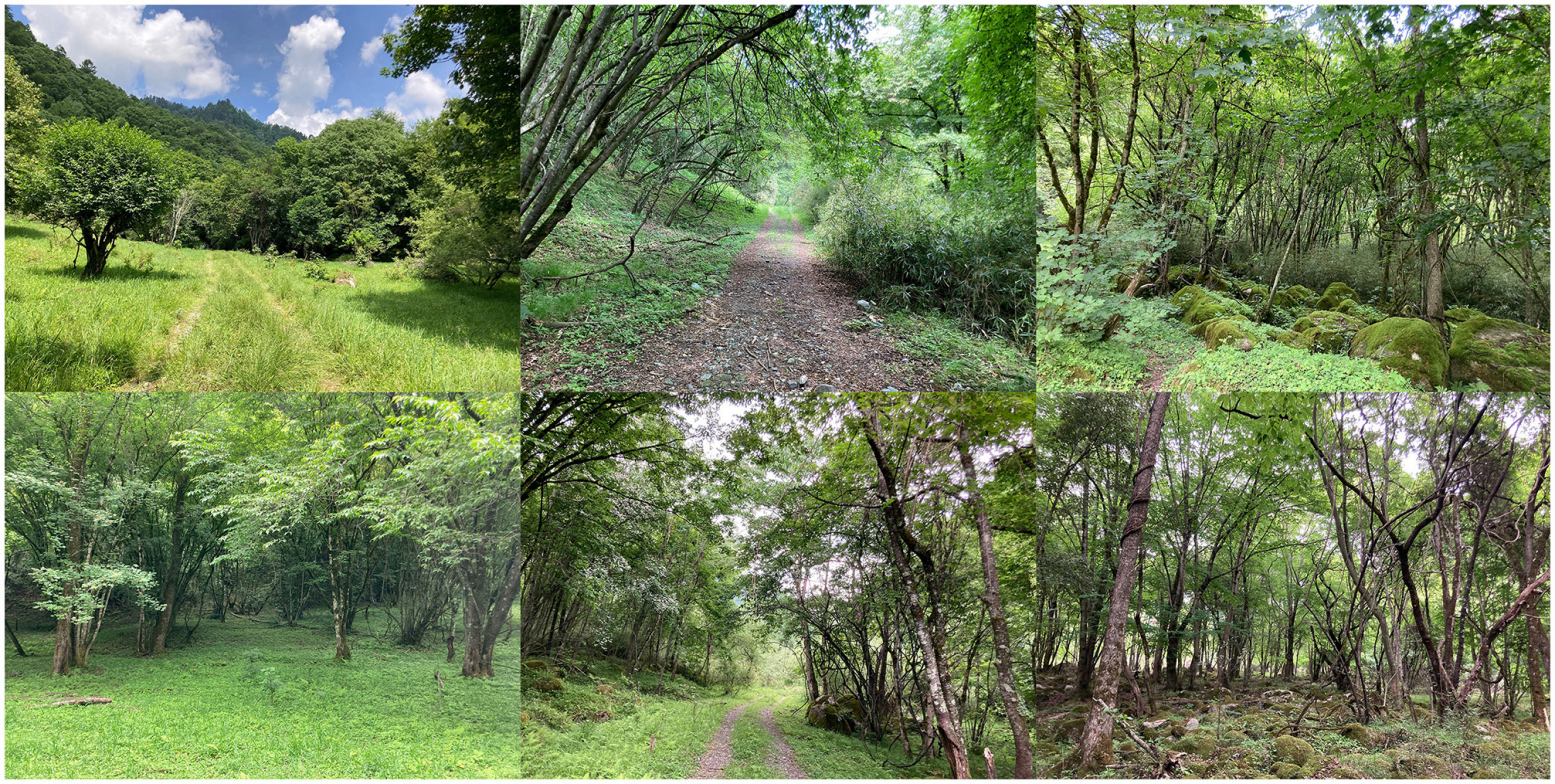
A pure natural environment for conducting FBN.

**Figure 3:**
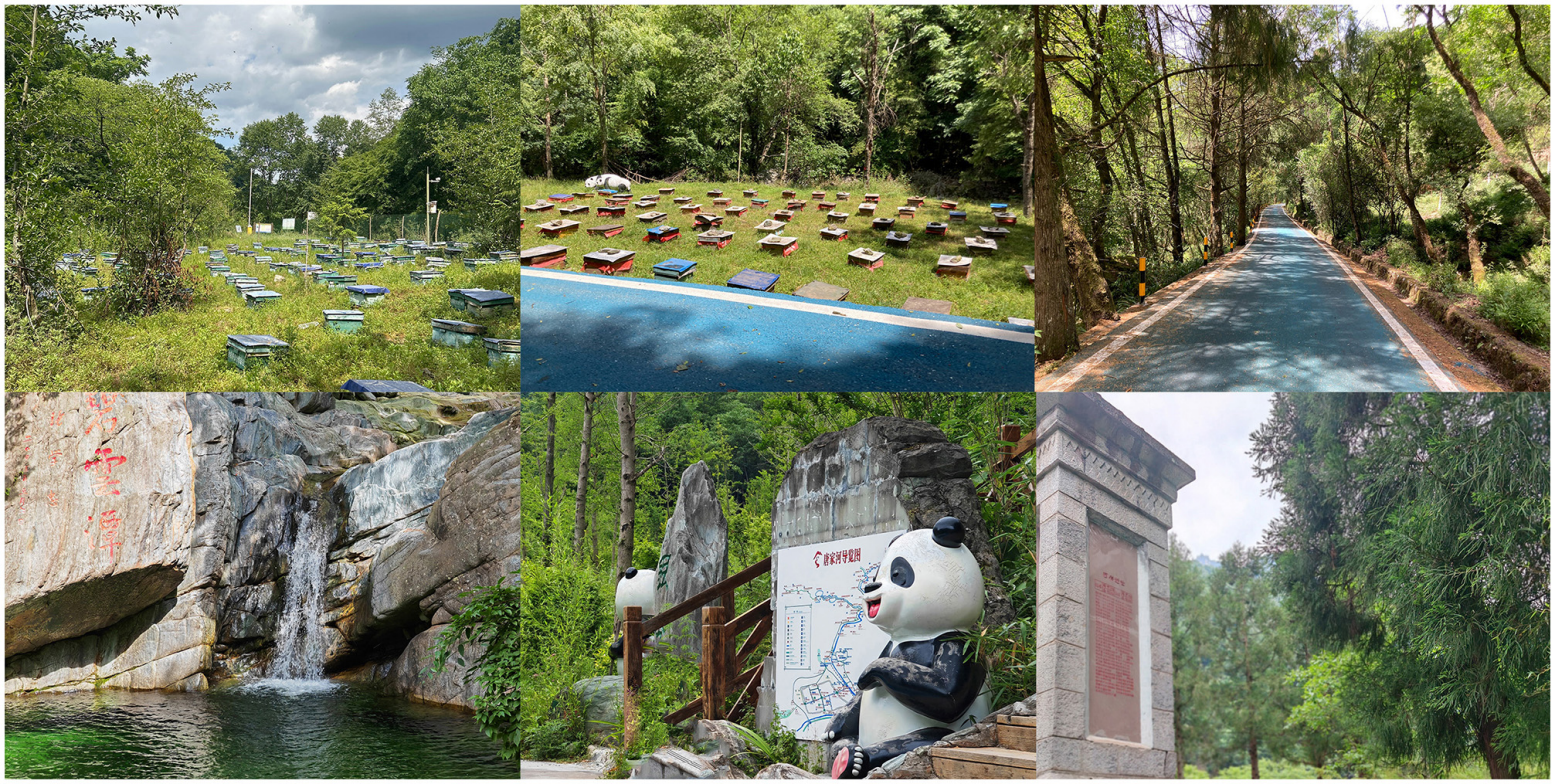
A natural environment with a cultural atmosphere for conducting FBC.

Forest bathing includes walking and breathing exercises. We selected natural scientific research and public tourism routes as experimental sites for forest bathing. Specifically, this included forest bathing in pure natural environments (FBN), and forest bathing in natural environments with cultural elements (FBC) (Figures 2, 3).

#### 2.2.2 Sensory therapy activity experimental sites

**Figure 4:**
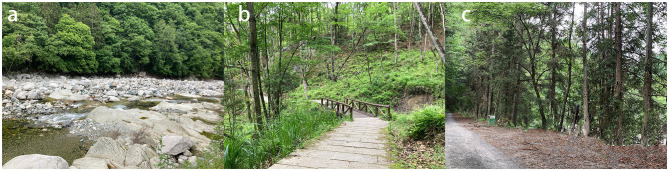
**(a)** A waterfront environment for conducting STW. **(b)** A forest environment for conducting STF. **(c)** Baduanjin Activity Site in STW.

Sensory therapy activities include sensory therapy in forest environments (STF) and sensory therapy by the water (STW). The experimental sites were relatively open, flat woodlands (sound pressure level 43.69 dBA ± 3.8, surrounding forest coverage over 95%) and water-side areas (75.22 dBA ± 0.8, approximately 1 m from water) (Figure 4).

### 2.3 Physiological and psychological health measurement indicators

Physiological feedback technology can objectively record changes in people's environmental perceptions and physiological states. Human health is a complex system, where neurotransmitters, respiratory circulation, immune systems, and bodily functions all play important roles in maintaining health (Babisch et al., 2001; Li et al., 2007, 2008; Seo et al., 2015; Thompson Coon et al., 2011). Therefore, physiological health measurements for participants in this study included heart rate (HR), blood pressure (BP), electroencephalography (EEG), catecholamine (3 items), lymphocyte subgroups (6 items), exhaled nitric oxide (FeNO), lung ventilation function, and physical fitness tests (including muscle strength, body flexibility, agility, and balance). Psychological health measurements included a revised version of the Self-Restore and Preference Scale (SRRPS), Sensory Assessment Scale (SAS), Leeds Sleep Evaluation Questionnaire (LSEQ), Transcendent Experience Scale (TES), and Personal Subjective WellBeing Index (PSWBI)reflecting perceived wellbeing.

#### 2.3.1 Physiological health measurement indicators

HR and BP are commonly used as indicators of physiological response. Previous research has widely confirmed that EEG can be used to measure brain activity. Different frequency bands reflect different emotional states and cognitive abilities. An increase in theta waves usually indicates a deeply relaxed state, which is associated with enhanced creativity (Schacter, 1977). Alpha waves are closely related to positive emotions and stress relief, with an increase typically indicating relaxation (Klimesch, 1999). An increase in beta waves is usually associated with alertness, while a decrease indicates drowsiness (Lee B. G. et al., 2014). The catecholamine system (3 items) includes dopamine (DA), norepinephrine (NE), and epinephrine (E), which are sympathetic neurotransmitters that influence both emotional responses and cardiovascular activity. Measurement of neurotransmitters can effectively analyze the effects of stimuli on receptors (Ramos and Mormède, 1997). The immune system plays a critical role in maintaining human health, with T cells, B cells, and natural killer (NK) cells being important immune cells. Analysis of T, B, and NK cells helps assess immune function levels and human health benefits (Li et al., 2007, 2008). The respiratory system plays a key role in normal life activities. The natural environment improves air quality and intercepts particles to reduce symptoms of acute respiratory diseases (Nowak et al., 2018). FeNO measurement reflects airway inflammation, with a decrease within the normal range indicating reduced small airway inflammation (Munakata, 2012). Lung ventilation measures respiratory airflow, lung volume, and gas exchange capabilities, with increases within the normal range indicating stronger respiratory capacity and lung function (Zhou and Zheng, 2012). Previous studies have shown that measuring respiratory-related indicators to assess the health and wellbeing benefits of natural ecosystems is both important and feasible (Seo et al., 2015). The characteristics of green spaces are closely related to physical activity and resident health (Thompson Coon et al., 2011). Specific measurement methods are outlined in Table 4.

**Table 4:** Physiological measurement items.

**Measurement items**		**Instrumentation**	**Methods**	**Reference interval value**
Circulatory system	Blood pressure (BP), heart rate (HR)	Omron upper arm electronic blood pressure monitor (HEM-8102a)	Wearable measurement method	90 mmHg ≤ systolic blood pressure (SBP) ≤ 140 mmHg 60 mmHg ≤ diastolic blood pressure (DBP) ≤ 90 mmHg 60–100 bpm (HR)
Respiratory system	Exhaled nitric oxide (FeNO)	Jinan runkai exhaled nitric oxide tester	Electrochemical detection method	5–30 ppb
	Lung function (maximum voluntary ventilation MVV, forced vital capacity FVC, FEV1/FVC)	Lung function tester (BH-AX-MAPG)	Differential pressure method	MVV>80%, FVC>80%, FEV1/FVC>70%
Neurotransmitter system	Catecholamines (dopamine DA, norepinephrine NE, epinephrine E)	AB Sciex 5,500+	HPLC method	DA (pg/mL) < 30.00 NE (pg/mL) reference range 200.00–1,700.00 (standing) E (pg/mL) ≤ 141.01(standing)
Immune system	Lymphocyte subpopulations NK cells (CD16+CD56)%, CD4/CD8 ratio, Helper T cells% (CD4+%), Suppressor/cytotoxic T cells% (CD8+%), total B lymphocytes (CD19)%, total T lymphocytes% (CD3+%)	Flow Cytometer (Mindray BriCyte E6)	Flow Cytometry	NK cells (CD16+CD56+): reference range 7.00–40.00% CD4/CD8 ratio: reference range 0.71–2.87 Helper T cells (CD4+%): reference range 27.00–51.00% Suppressor/cytotoxic T cells (CD8+%): reference range 15.00–44.00% Total B lymphocytes (CD19+%): reference range 5.00–18.00% Total T lymphocytes (CD3+%): Reference range 50.00–84.00%
	Brain waves (theta waves, alpha waves, beta waves)	Huimind brain instrument (sichiray03)	Wearable measurement method	The value increases after the experiment.
Physical fitness test	Muscle strength (push-ups)	None	Counting method	The value increases after the experiment.
	Flexibility (sit and reach)	Sit and reach tester	Measurement method	The value increases after the experiment.
	Balance (one-leg stand)	Timer	Timing method	The value increases after the experiment.
	Agility (T-agility test)	Timer	Timing method	The value decreases after the experiment

In this study, the method for evaluating muscle strength in the physical fitness test is the number of push-ups completed with maximum effort in a single attempt, using the counting method. Flexibility is measured as the length of the sit and reach, using the measurement method, unit: cm. Balance is measured by the duration of standing on one leg, using the timing method, unit: seconds. Agility is measured by the duration of the T-Agility Test, using the timing method, unit: seconds (Li et al., 2008).

#### 2.3.2 Psychological health measurement indicators

Psychological health measurements in this study are shown in Table 5 Based on prior research, a self-report measure was developed to assess the restorative potential of different landscape environments across four dimensions: restorative experience, positive emotions, stress relief, and physiological-cognitive responses (Thomas and Palma, 2018). The revised version of the SRRPS (Han, 2003) includes five dimensions: emotions, physiology, cognition, behavior, and aesthetic preferences, with a total of 9 items, each rated on a 1–5 scale (5 indicating the best psychological restoration effect and 1 indicating the worst). The total value of Cronbach's α coefficient for the scale is 0.894. The PSWBI (Cummins et al., 2003) includes seven dimensions: living standard, health, achievement, safety, connection, future security, and interpersonal relationships, with a total of 7 items, each rated on a 1–10 scale (10 indicating the best level of wellbeing effect and 1 indicating the worst). The total value of Cronbach's α coefficient for the scale is 0.960. The TES (Ballew and Omoto, 2018; Kaplan and Kaplan, 1989) includes 3 dimensions: humility, awe, and reflection, with a total of 3 items, each rated on a 1–5 scale (5 indicating the best experience and 1 indicating the worst). The total value of Cronbach's α coefficient for the scale is 0.800. In addition, a (SAS Park et al., 2007) was added, which includes two dimensions: calmness and comfort, with a total of 2 items, each rated on a 1–13 scale (13 indicating the highest level of sensory assessment effects and 1 indicating the lowest level). The total value of Cronbach's α coefficient for the scale is 0.926.

**Table 5:** Psychological measurements and questionnaire items.

**Scale name**	**Measurement indicators**	**Scoring rating**	**Cronbach's alpha**	**Number of items**
Shortened recovery and preference scale (SRRPS)	Mood	Scored on a 1–5 scale	0.894	9
	Physiological			
	Cognitive			
	Behavior			
	Aesthetic preference			
Personal subjective wellbeing index (PSWBI)	Living standards	Scored on a 1–10 scale	0.960	7
	Health			
	Achievement			
	Safety			
	Connectivity			
	Future security			
	Interpersonal relationships			
Transcendent experience scale (TES)	Humility	Scored on a 1–5 scale	0.800	3
	Awe			
	Reflection			
Sensory assessment scale (SAS)	Calmness	Scored on a 1–13 scale	0.926	2
	Comfort			
Leeds sleep evaluation questionnaire (LSEQ)	Sleep onset (GTS)	Scored on a 1–10 scale	0.949	10
	Sleep quality (QOS)			
	Awakening (AFS)			
	Post-waking behavior (BFW)			

#### 2.3.3 Sleep quality questionnaire measurement

In this study, the LSEQ was used to assess sleep quality (Table 5), including four dimensions: sleep onset (GTS), sleep quality (QOS), awakening (AFS), and post-awakening behavior (BFW), with a total of 10 items (Thomas and Palma, 2018). In GTS, each subitem rated on a 1–10 scale (10 indicating the easiest to fall asleep and 1 indicating the hardest). In QOS, each subitem rated on a 1–10 scale (10 indicating the best sleep quality and 1 indicating the worst). In AFS, each subitem rated on a 1–10 scale (10 indicating the highest number of times waking up at night or waking up early, and 1 indicating the lowest). In BFW, each subitem rated on a 1–10 scale (10 indicating the best state upon waking and 1 indicating the worst). The LSEQ can assess sleep quality through short-term measurement, making it suitable for this study. The total value of Cronbach's α coefficient for the scale is 0.949.

## 3 Experimental process

The experiment was conducted in the Tangjiahe section of the GPNP from June 11 to 15, under favorable weather conditions. During June 11–15, the average environmental temperature along two routes ranged from 16°C to 23°C, humidity ranged from 68 to 81% RH, PM2.5 ranged from 4 to 12 μg/m3, and negative oxygen ions ranged from 5,478.00 to 6,695.58/cm3 (Tables 2, 3). 32 participants were randomly assigned experimental numbers from 1 to 32, divided into four groups (1, 2, 3, and 4) of eight participants each. Groups 1 and 2 were tested from June 11 to 13, while groups 3 and 4 were tested from June 13 to 15. The experimental process for groups 1 and 3 was identical to that of groups 2 and 4, conducted simultaneously to eliminate time differences‘ effects. Researchers only informed participants of the experimental purpose without disclosing detailed procedures, and encouraged them to walk leisurely in the forest while engaging in multisensory perception. During the experiment, participants practiced forest bathing and completed sensory therapy activities at designated sites. These activities, including observing flora and fauna, listening to flowing water, inhaling forest air, and tree hugging, aimed to facilitate self-forgetfulness and foster interaction with nature within the national park's forest environment. Numerous photographs taken by accompanying staff documented participants' relaxed states while avoiding potential intervention risks.

**Day 1 (June 11/13)** At 8:00 AM, 32 participants and staff departed from Sichuan Forestry Central Hospital by bus to the Tangjiahe section of the park. Before departure, staff briefed participants on the experiment's purpose, process, and precautions, and guided them to fill out the previous night's LSEQ. Upon arrival, participants completed the first baseline physiological measurements, including EEG, three catecholamines, six lymphocyte subpopulation tests, FeNO, three lung ventilation functions, HR, and BP. Afterward, participants completed the first physical fitness test under staff guidance. Participants stayed overnight at the park's Senyang Villa Research Center.

**Day 2 (June 12/14)** At 7:30 AM, participants filled out the previous night's LSEQ. Staff then guided participants in group activities for 3 h (8:30 to 11:30 AM) at their respective experimental sites. Groups 1 and 3 engaged in FBN, while groups 2 and 4 engaged in STW. During the activities, participants recorded CES perceived values. The experiments at both research sites were conducted simultaneously. In FBN, staff guided participants on a combined walking and bus (experimental tour bus) journey along the experimental route, with a total of 30 min of bus rides and 90 min of walking, including breaks, completing 15-min respiratory function activities. In STW, participants engaged in 30 min of mindfulness meditation, 90 min of sensory activities (smell, taste, and touch), including plant mandalas and tree hugging, and 30 min of Baduanjin exercises. After the activities, participants returned collectively (travel time 30 min), rested for 10 min, and then completed the second set of physiological measurements and physical fitness tests, followed by psychological scales and CES value assessments. The second day's experiment concluded.

**Day 3 (June 13/15)** At 7:30 AM, participants filled out the LSEQ. Groups 1 and 3 conducted STF, while groups 2 and 4 conducted FBC. Data collection before and after the activities was consistent with Day 2.

**Figure 5:**
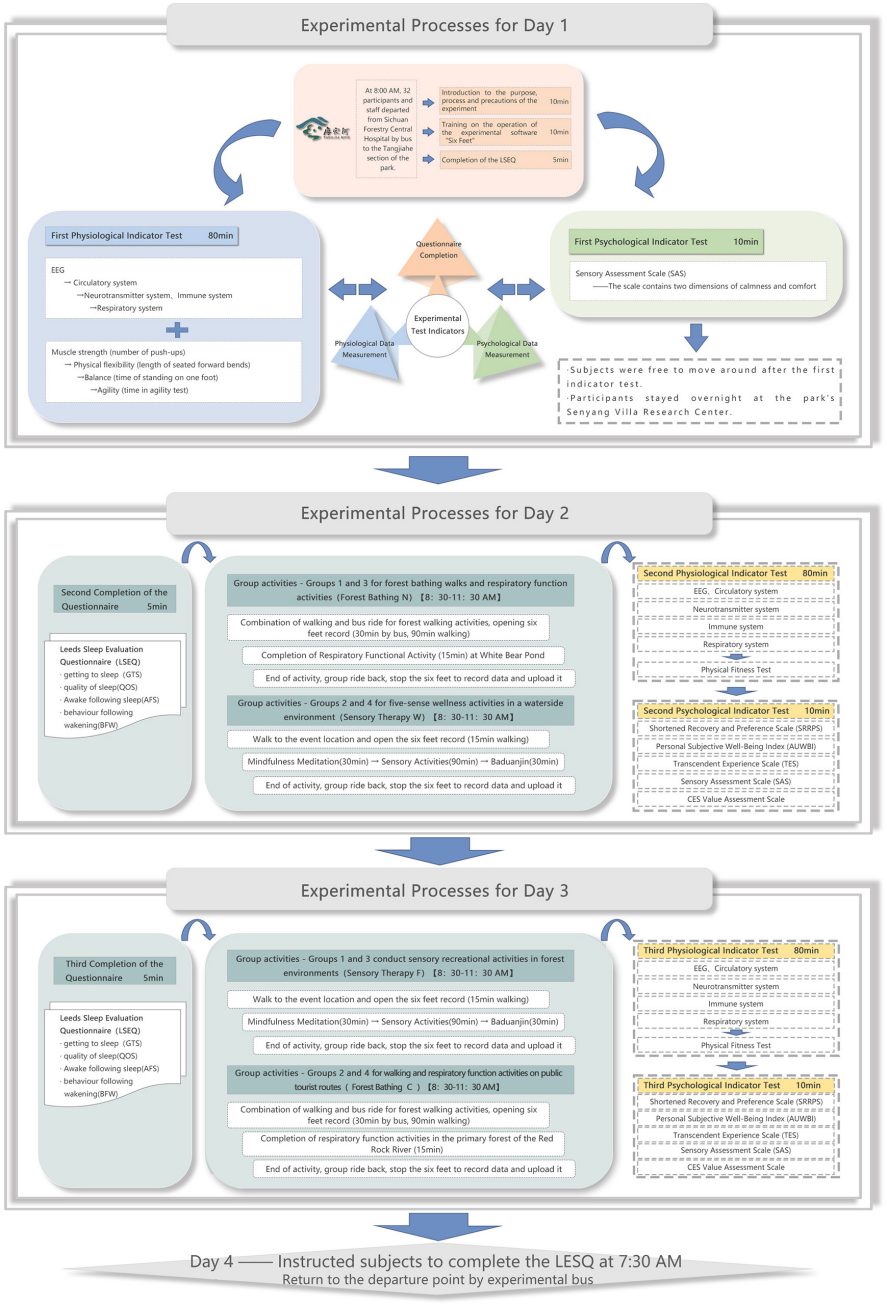
Experimental flowchart. Participants had standardized diets and routines during the experiment, consistent with their daily habits, without special adjustments.

**Figure 6:**
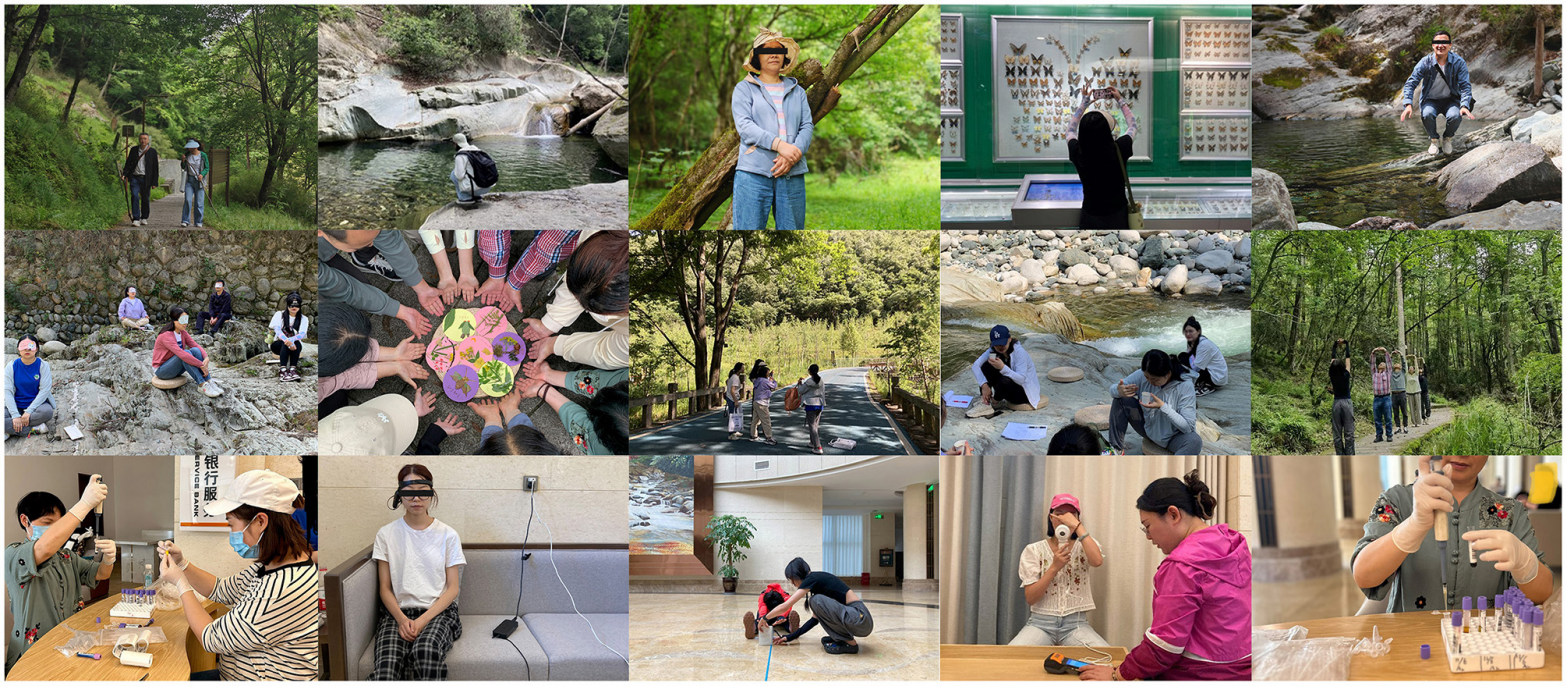
Experimental flowchart.

**Day 4** At 7:30 AM, participants filled out the LSEQ, marking the end of the experiment. After completing the data collection within the park concluded, participants and staff returned by the experimental bus (Figures 5, 6).

## 4 Statistical analysis

The sample size for the experiment was 32. This study employed a relatively comprehensive set of indicators, primarily aiming to explore the physiological and psychological health benefits experienced by participants after undergoing therapy experiments in national parks, specifically analyzing pre- and post-experiment differences. To verify whether the data conformed to a normal distribution, we conducted the Shapiro Wilk test on all sample data. The results showed that the *p*-values were all greater than 0.05. Therefore, we accepted the null hypothesis and concluded that the data from the experimental conformed to a normal distribution. Based on this, data processing was conducted using SPSS 26.0 software (SPSS Inc., Chicago, IL, USA). Paired *t*-tests were employed to compare the mean differences between post-experiment physiological and psychological indicators and their pre-experiment baseline values. The LSEQ was scored using paired *t*-tests, incorporating both arithmetic sum and average. Psychological data were scored using arithmetic sum and average. In this study, a *P*-value < 0.05 indicated statistical significance. Psychological data, due to different scale levels, were normalized first, with the normalized value = (original value–minimum possible value)/(maximum possible value–minimum possible value).

## 5 Results

### 5.1 Sociodemographic characteristics

Thirty-two subjects participated in the study. They all felt tired from time to time, had high work pressure, and had no recent sudden experiences of happier or sadder things. Notably, they were asked how often they visit the landscape. Approximately 90% of them visited once a month or once every few months, 6% visited a few times a month, and 4% hardly ever. There were no significant differences in the above sociodemographic characteristics.

### 5.2 Analysis of physiological health benefits of subject health care workers after forest therapy in GPNP

#### 5.2.1 EEG data analysis

**Figure 7:**
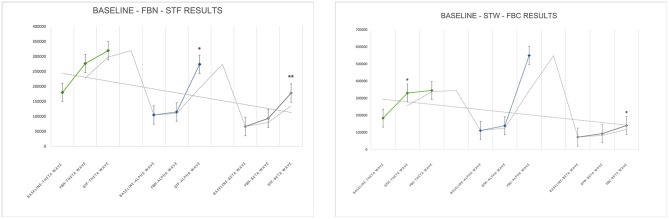
EEG test results. *N* = 32. *p < 0.05, **p < 0.01.

Compared to baseline values, there were no statistically significant differences in theta, alpha, and beta wave values after FBN (*P* > 0.05), though an upward trend was observed. After STF, alpha (pre-experiment to post-experiment is [1,04,916.53 ± 1,19,003.17] to [2,73,893.28 ± 2,47,930.68]) and beta ([66,260.05 ± 75,511.02] to [1,77,799.69 ± 1,10,698.34]) wave values showed statistically significant differences (*P* < 0.05). Specifically, theta wave increased after FBN and STF were 96,715 and 1,39,593, respectively. Alpha wave values increased by 9,823 and 1,68,976 after FBN and STF, respectively, with the STF increase showing significant difference ([1,04,916.53 ± 1,19,003.17] to [2,73,893.28 ± 2,47,930.68]; *P* = 0.011). Beta wave values increased by 27,018 and 111,539 after FBN and STF, respectively, with the STF increase showing significant difference ([66,260.15 ± 75,511.02] to [1,77,799.69 ± 1,10,698.34]; *P* = 0.005). Compared to baseline values, theta, alpha, and beta wave values increased after both FBC and STW. Specifically, theta wave values increased by 146,808 after STW ([1,83,408.14 ± 1,77,499.73] to [3,30,216.71 ± 1,57,293.53]; *P* = 0.019), while beta wave values increased by 67,483 after FBC ([72,958.75 ± 71,516.33] to [1,40,442.67 ± 89,273.58]; *P* = 0.05), both showing statistically significant differences (Figure 7).

#### 5.2.2 Neurotransmitter system and immune system data analysis

**Figure 8:**
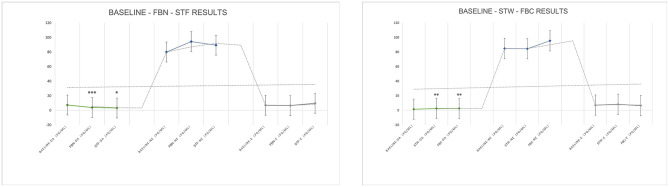
Neurotransmitter system catecholamine test results. *N* = 32. *p < 0.05, **p < 0.01, ***p < 0.001.

**Figure 9:**
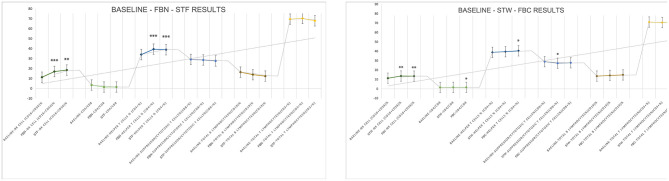
Immune system lymphocyte subpopulation test results. *N* = 32. *p < 0.05, **p < 0.01, ***p < 0.001.

Among the three catecholamine indices, DA levels increased by 1.764 pg/mL and 1.178 pg/mL compared to baseline values after FBN and STF, respectively, both showing significant differences ([2.04 ± 1.12] to [3.80 ± 2.53], *P* = 0.001; [2.04 ± 1.12] to [3.21 ± 2.43], *P* = 0.028). After STF, E levels increased by 3.10 pg/mL. After STW and FBC, DA levels increased by 0.95 and 1.00 pg/mL, respectively, both showing significant differences ([1.49 ± 0.61] to [2.44 ± 1.21], *P* = 0.006; [1.49 ± 0.61] to [2.49 ± 1.24], *P* = 0.006). After FBN and STF, among lymphocyte subsets indices, the NK cells (CD16 + CD56)% remained within the normal range (reference range 7.00–40.00%), showing increases of 5.705 ([11.32 ± 6.37] to [17.03 ± 8.58], *P* = 0.001) and 7.142 ([11.32 ± 6.37] to [18.47 ± 10.44], *P* = 0.002) compared to baseline, both representing significant differences. The helper T cells (CD4+%) increased within the normal reference range (reference range 27.00–51.00%) by 5.420 ([34.04 ± 5.17] to [39.46 ± 4.78], *P* < 0.001) and 4.871 ([34.04 ± 5.17] to [38.91 ± 5.29], *P* = 0.001), both showing significant differences. After STW and FBC, the NK cells (CD16 + CD56)% increased within the normal range by 2.30 ([11.31 ± 4.36] to [13.62 ± 5.45], *P* = 0.005) and 2.16 ([11.31 ± 4.36] to [13.48 ± 5.38], *P* = 0.005), respectively, representing significant differences. After FBC, the CD4/CD8 ratio increased by 0.96 ([1.44 ± 0.50] to [1.59 ± 0.65], *P* = 0.035) within the normal range (0.71–2.87), representing a significant difference. The helper T cells (CD4+%) increased by 1.64 within the normal range ([38.83 ± 5.16] to [40.47 ± 5.84], *P* = 0.026), showing significant difference. After STW, the Suppressor/Cytotoxic T cells (CD8+%) rose by 1.650 ([28.93 ± 6.41] to [27.28 ± 7.06], *P* = 0.024), demonstrating significant difference. Other indicators showed no significant differences but exhibited upward trends. Furthermore, forest bathing and sensory therapy in different environments exerted significant positive effects on DA levels and demonstrated significant effects on regulating positive emotions. Results indicate that implementing forest bathing and sensory therapy activities in diverse environments within the GPNP significantly enhances human immune function, particularly increasing NK cells counts (Figures 8, 9).

#### 5.2.3 Respiratory and circulatory system data analysis

**Figure 10:**
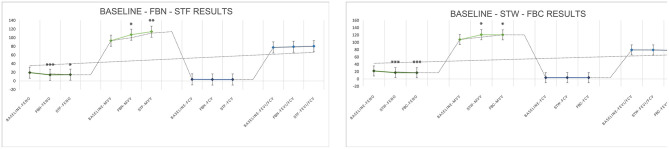
Respiratory (FeNO, MVV, FVC, FEV1/FVC) test results. *N* = 32. *p < 0.05, **p < 0.01, ***p < 0.001.

**Figure 11:**
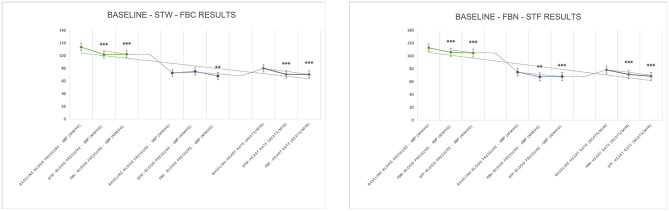
BP and HR test results. *N* = 32. *p < 0.05, **p < 0.01, ***p < 0.001.

After FBN, FBC, STF, and STW, FeNO values decreased significantly within the normal range (reference range 5–30 ppb) by 5.07 ppb ([19.29 ± 4.29] to [14.21 ± 2.91], *P* < 0.001), 4.786 ppb ([22.00 ± 9.44] to [17.21 ± 9.38], *P* < 0.001), 4.71 ppb ([19.29 ± 4.29] to [14.57 ± 2.98], *P* = 0.007), and 4.571 ppb ([22.00 ± 9.44] to [17.43 ± 9.16], *P* < 0.001), respectively. MVV increased within the normal range (reference range MVV > 80%) by 13.90 ([93.15 ± 23.66] to [107.05 ± 20.22], *P* = 0.022), 12.88 ([107.76 ± 32.56] to [120.64 ± 27.91], *P* = 0.028), 20.9 ([93.15 ± 23.66] to [114.05 ± 18.42], *P* = 0.006), and 13.12 ([107.76 ± 32.56] to [120.88 ± 37.99], *P* = 0.027), respectively, all showing significant differences. Regarding BP and HR, all indicators decreased significantly except for DBP, which increased after STW. Specifically, after FBN and STF, SBP decreased by 7.00 mmHg ([112.71 ± 11.33] to [105.71 ± 11.79], *P* < 0.001) and 8.07 mmHg ([112.71 ± 11.33] to [104.64 ± 11.17], *P* < 0.001), respectively, while DBP decreased by 7.29 mmHg ([74.86 ± 7.19] to [67.57 ± 6.47], *P* = 0.002) and 6.71 mmHg ([74.86 ± 7.19] to [68.14 ± 9.73], *P* < 0.001), respectively, all showing significant differences. After STW and FBC, SBP decreased by 11.64 mmHg ([113.43 ± 12.79] to [101.79 ± 12.36], *P* < 0.001) and 11.14 mmHg ([113.43 ± 12.79] to [102.29 ± 12.00], *P* < 0.001), respectively, both showing significant differences. DBP decreased by 4.79 mmHg ([72.64 ± 8.60] to [67.86 ± 7.41], *P* = 0.005) after FBC, while it slightly increased by 2.42 mmHg ([72.64 ± 8.60] to [75.07 ± 29.77], *P* = 0.76) after STW, with no significant difference. After FBN, FBC, STF, and STW, HR decreased by 7.07 bpm ([78.29 ± 8.25] to [71.21 ± 8.19], *P* = 0.001), 9.64 bpm ([79.93 ± 12.30] to [70.29 ± 11.06], *P* < 0.001), 9.86 bpm ([78.29 ± 8.25] to [68.43 ± 7.07], *P* < 0.001), and 9.43 bpm ([79.93 ± 12.30] to [70.50 ± 11.57], *P* < 0.001), respectively, all showing significant differences. Results indicate that forest bathing and sensory therapy activities conducted in diverse environments within the GPNP deliver significant calming and relaxation health benefits for urban healthcare workers (Figures 10, 11).

#### 5.2.4 LSEQ data analysis

##### 5.2.4.1 Comparative analysis between the night before departure and the arrival night, FBN night, and STF night (return night)

**Figure 12:**
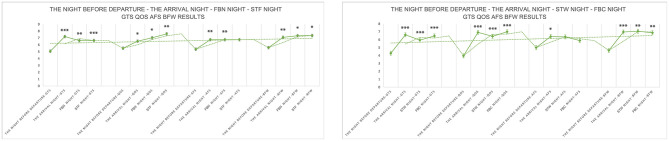
LSEQ (GTS, QQS, AFS, BFW) test results. *N* = 32. *p < 0.05, **p < 0.01, ***p < 0.001.

**Figure 13:**
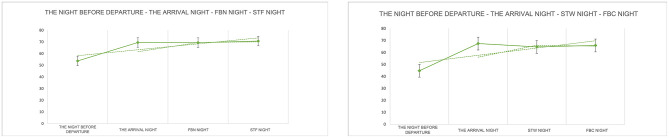
LSEQ scoring results.

Compared to the night before departure, GTS significantly increased on the arrival night, FBN night, and STF night (return night), with respective increases of 2.14 ([5.07 ± 1.85] to [7.21 ± 0.85], *P* = 0.001), 1.55 ([5.07 ± 1.85] to [6.12 ± 1.71], *P* = 0.002), and 1.57 ([5.07 ± 1.85] to [6.64 ± 1.66], *P* < 0.001). QQS values showed significant increases, with respective increases of 1.04 ([5.50 ± 2.72] to [6.54 ± 2.13], *P* = 0.016), 1.54 ([5.50 ± 2.72] to [7.04 ± 2.21], *P* = 0.021), and 2.11 ([5.50 ± 2.72] to [7.60 ± 2.01], *P* = 0.008). No significant differences were observed between the arrival night, FBN night and STF night. AFS values significantly increased on the arrival night and FBN night, at 1.39 ([5.36 ± 2.48] to [6.75 ± 1.84], *P* = 0.003) and 1.39 ([5.36 ± 2.48] to [6.75 ± 2.39], *P* = 0.009), respectively. STF night also showed an increase, but it was not significant. BFW values also significantly increased 1.50 ([5.60 ± 2.32] to [7.10 ± 2.08], *P* = 0.003), 1.74 ([5.60 ± 2.32] to [7.33 ± 1.96], *P* = 0.017), and 1.76 ([5.60 ± 2.32] to [7.36 ± 2.14], *P* = 0.006). No significant differences existed among the four measures on arrival night, FBN night, and STF night (Figure 12). The mean LSEQ total score on the night before departure was 53.7, indicating fair sleep quality. On arrival night, FBN night, and STF night (return night), the mean LSEQ total scores were 69.5, 69.4, and 70.7, indicating fair sleep quality (Figure 13).

##### 5.2.4.2 Comparative analysis between the night before departure and the arrival night, STW night, and FBC night (return night)

Compared to the night before departure, GTS significantly increased on the arrival night, STW night, and FBC night (return night), with increases of 2.33 ([4.29 ± 1.04] to [6.62 ± 0.99], *P* < 0.001), 1.67 ([4.29 ± 1.04] to [5.95 ± 1.35], *P* < 0.001), and 2.19 ([4.29 ± 1.04] to [6.48 ± 1.29], *P* < 0.001), respectively. QOS values showed significant increases, with respective increases of 2.96 ([3.96 ± 1.41] to [6.93 ± 1.55], *P* < 0.001), 2.46 ([3.96 ± 1.41] to [6.43 ± 1.89], *P* < 0.001), and 3.04 ([3.96 ± 1.41] to [7.00 ± 1.52], *P* < 0.001). AFS values significantly increased 1.39 on the arrival night ([5.00 ± 1.92] to [6.93 ± 1.38], *P* = 0.023), while STW and FBC nights showed increases that were not statistically significant. BFW values also significantly increased, with respective increases of 2.33 ([4.64 ± 2.07] to [6.98 ± 1.50], *P* < 0.001), 2.43 ([4.64 ± 2.07] to [7.07 ± 2.28], *P* = 0.002), and 2.24 ([4.64 ± 2.07] to [6.88 ± 1.94], *P* = 0.002) (Figure 12). The average LSEQ total score on the night before departure was 44.7, indicating poor sleep quality. On the arrival night, STW night and FBC night (return night), the average LSEQ total scores were 67.4, 64.6, and 65.9, respectively, indicating better sleep quality. Baseline data revealed overall poor sleep quality among urban healthcare workers due to high-stress occupations. Experimental results indicate that forest bathing and sensory therapy activities within the GPNP significantly improve sleep onset difficulties and sleep quality. Notably, the forest environment of the GPNP exert a greater positive impact on sleep (Figure 13).

#### 5.2.5 Physical fitness data analysis

**Figure 14:**
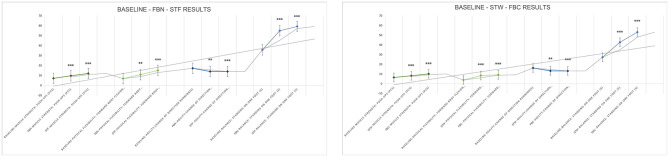
Physical fitness data analysis. *N* = 32. *p < 0.05, **p < 0.01, ***p < 0.001.

Regarding physical fitness (capacity building), all metrics showed significant improvements after FBN and STF compared to respective baseline values. Specifically: muscle strength (push-ups: reps) increased by 2.57 ([7.14 ± 5.63] to [9.71 ± 7.12], *P* = 0.001) and 4.93 ([7.14 ± 5.63] to [12.07 ± 7.48], *P* < 0.001). Flexibility (sit and reach: cm) increased by 3.93 ([6.93 ± 8.90] to [10.86 ± 2.59], *P* = 0.010) and 8.14 ([6.93 ± 8.90] to [15.07 ± 9.64], *P* < 0.001). Agility (shuttle run: s) decreased by 3.22 ([17.23 ± 3.92] to [14.01 ± 1.35], *P* = 0.002) and 3.22 ([17.23 ± 3.92] to [14.01 ± 1.43], *P* = 0.001). Balance (one-leg stand: s) increased by 19.21 ([35.71 ± 18.69] to [54.93 ± 22.39], *P* < 0.001) and 23.50 ([35.71 ± 18.69] to [59.21 ± 25.67], *P* < 0.001). Significant improvements were observed after both STW and FBC. Specifically: muscle strength (push-ups: reps) increased by 1.71 ([6.50 ± 4.15] to [8.21 ± 4.49], *P* < 0.001) and 3.64 ([6.50 ± 4.15] to [10.14 ± 5.14], *P* < 0.001). Flexibility (sit and reach: cm) increased by 4.57 ([3.86 ± 9.88] to [8.43 ± 9.23], *P* = 0.001) and 5.29 ([3.86 ± 9.88] to [9.14 ± 9.00], *P* < 0.001). Agility (shuttle run: s) decreased by 2.99 ([16.21 ± 3.60] to [13.21 ± 1.11], *P* = 0.004) and 3.06 ([16.21 ± 3.60] to [13.14 ± 1.53], *P* = 0.001). Balance (one-leg stand: s) increased by 15.43 ([27.29 ± 10.82] to [42.71 ± 11.85], *P* < 0.001) and 25.79 ([27.29 ± 10.82] to [53.07 ± 11.72], *P* < 0.001) (Figure 14). The results indicate that forest bathing and sensory therapy activities in diverse environments within the GPNP significantly enhance physical fitness.

### 5.3 Analysis of the results of the impact of cultural ecosystem service (CES) values obtained by urban healthcare workers in different environments of the GPNP on psychological wellbeing and individual subjective perceptions of wellbeing

#### 5.3.1 The environment for FBN

**Figure 15:**
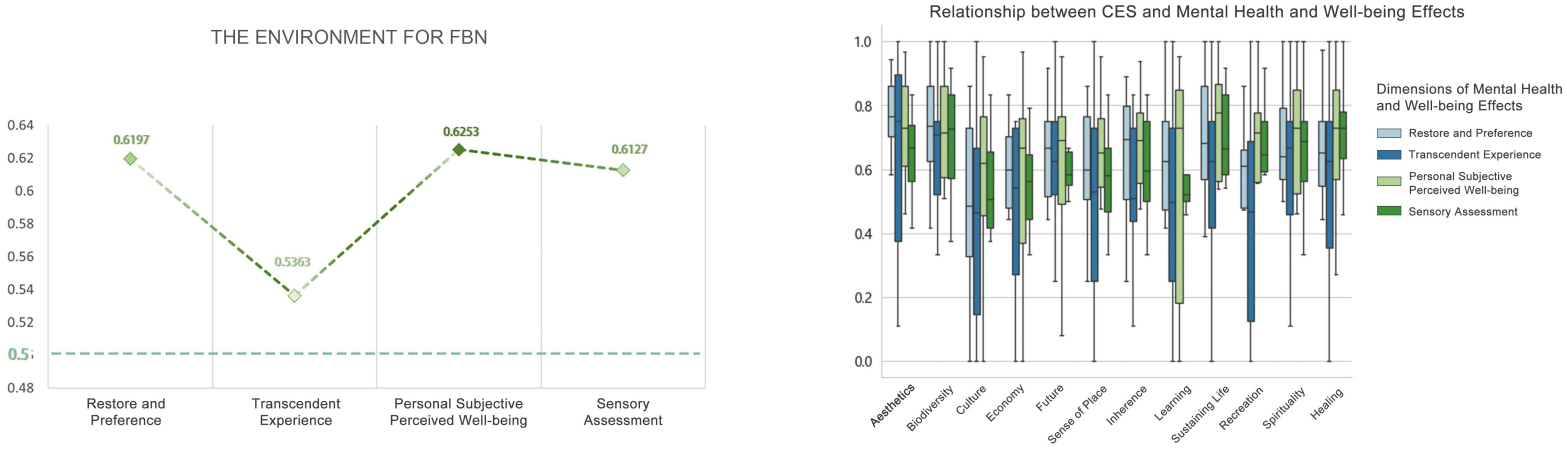
Relationship between CES and mental health and wellbeing effects (the environment for FBN).

The overall arithmetic mean distribution of each dimension for urban healthcare workers in the FBN ranged from 0.54 to 0.63, which were all greater than 0.5. Among them, the results showed significant differences between Restore and Preference and Transcendent Experience, and Transcendent Experience were significantly different from Personal Subjective Perceived Wellbeing and Sensory Assessment. Among them, the top three CES values that received Restore and Preference, Transcendent Experience, Personal Subjective Perceived Wellbeing, and Sensory Assessment effect pointers, respectively, were: aesthetics (0.7778), biodiversity (0.7440), sustaining life (0.6766); biodiversity (0.6474), aesthetics (0.6496), spirituality (0.6303); biodiversity (0.7002), aesthetics (0.6958), healing (0.6762); and biodiversity (0.7019), aesthetics (0.6506), sustaining life (0.6635) (Figure 15).

#### 5.3.2 The environment for FBC

**Figure 16:**
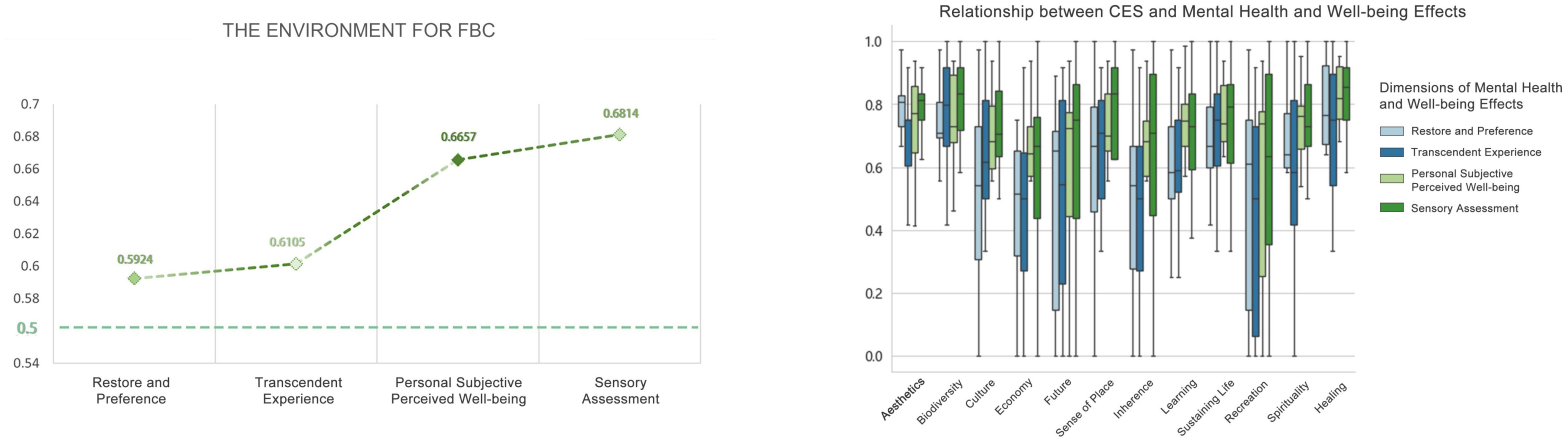
Relationship between CES and mental health and wellbeing effects (the environment for FBC).

The overall arithmetic means for each dimension were distributed between 0.59 and 0.68, all greater than 0.5. There were no statistically significant differences in effects between dimensions. The top three CES values that produced Restore and Preference, Transcendent Experience, Personal Subjective Perceived Wellbeing, and Sensory Assessment were all aesthetics (0.7540, 0.7115, 0.7347, 0.7724), biodiversity (0.7222, 0.7628, 0.7404, 0.7917), and healing (0.7560, 0.7179, 0.7880, 0.7981) (Figure 16).

#### 5.3.3 The environment for STF

**Figure 17:**
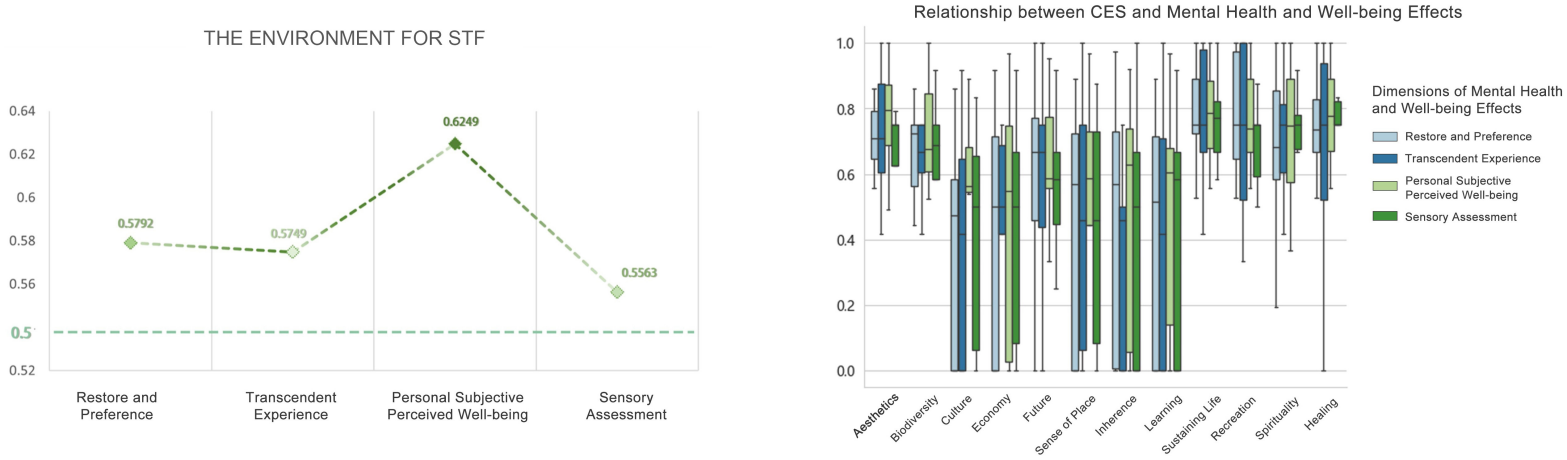
Relationship between CES and mental health and wellbeing effects (the environment for STF).

The overall arithmetic means for each dimension were distributed between 0.57 and 0.66, all greater than 0.5. There were no statistically significant differences in the dimension effects. The top three CES values to which Restore and Preference, Transcendent Experience, Personal Subjective Perceived Wellbeing, and Sensory Assessment effects pointed, respectively, were sustaining life (0.7798), recreation (0.7738), aesthetics (0.7143); sustaining life (0.7679), recreation (0.7321), spirituality (0.7262), aesthetics (0.7262); sustaining life (0.7834), aesthetics (0.7789), recreation (0.7732); and sustaining life (0.7143), healing (0.7113), spirituality (0.6756) (Figure 17).

#### 5.3.4 The environment for STW

**Figure 18:**
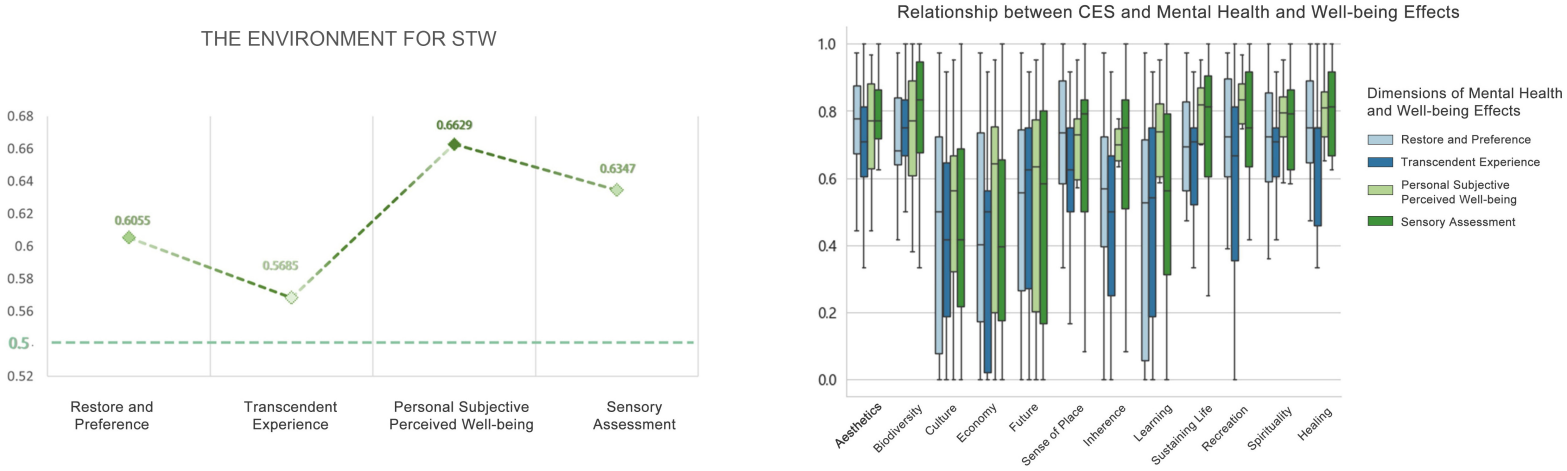
Relationship between CES and mental health and wellbeing effects (the environment for STW).

The overall arithmetic means for each dimension were distributed between 0.57 and 0.66, all greater than 0.5. There were no statistically significant differences in the dimension effects. The top three CES values to which Restore and Preference, Transcendent Experience, Personal Subjective Perceived Wellbeing, and Sensory Assessment effects pointed, respectively, were aesthetics (0.7679), sense of place (0.7242), healing (0.7183); biodiversity (0.7321), aesthetics (0.7143), spirituality (0.6786); recreation (0.7800), spirituality (0.7732), aesthetics (0.7517); and biodiversity (0.7946), aesthetics (0.7649), spirituality (0.7351) (Figure 18).

### 5.4 Analysis of the results of the impact of urban healthcare workers' recreational activities in different environments of the GPNP on mental health and individual subjective perceived wellbeing

#### 5.4.1 Activities in forest bathing environment

**Figure 19:**
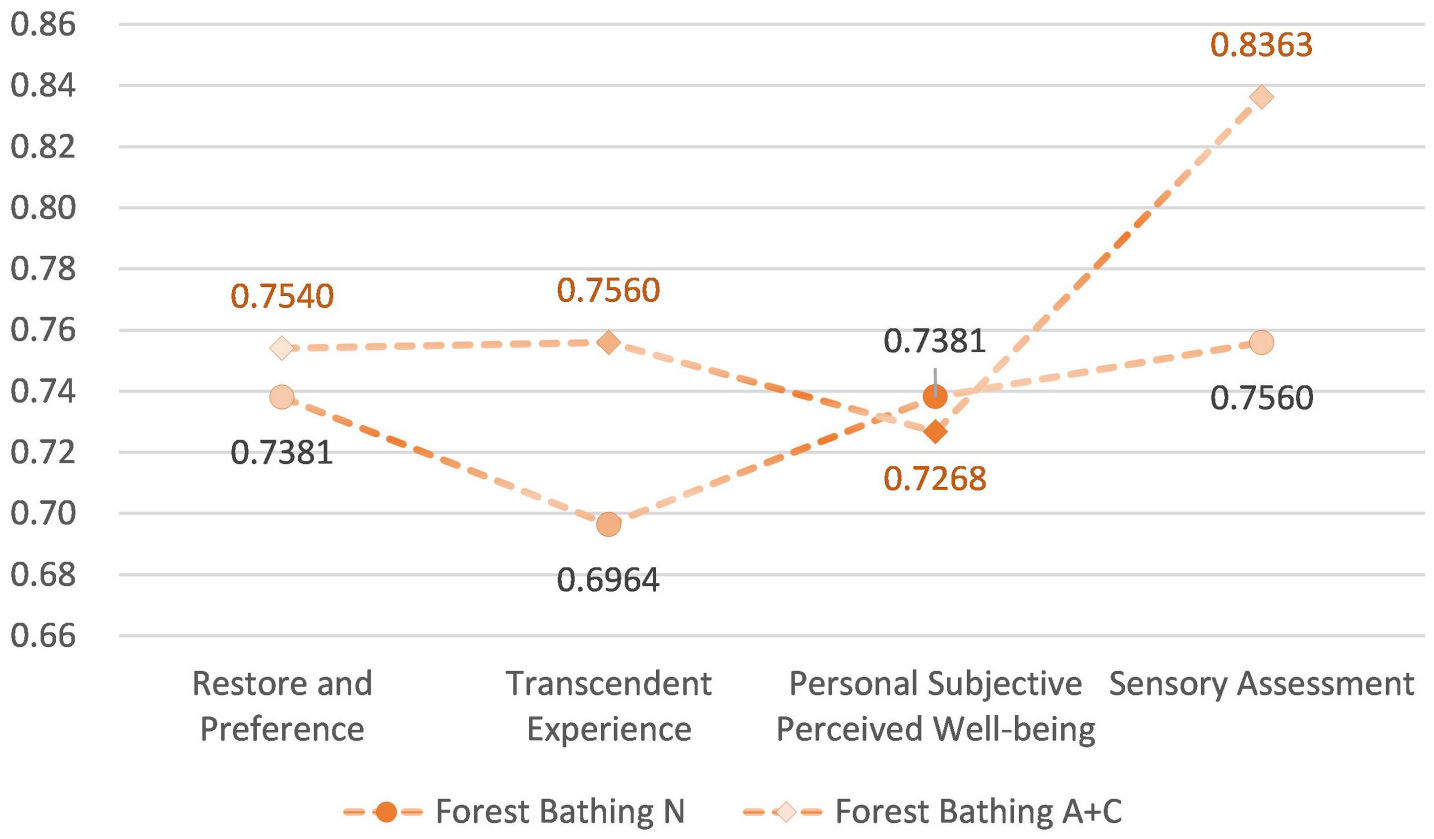
Overall mean distribution of the four dimensions of mental health and wellbeing effects from forest bathing activities.

**Figure 20:**
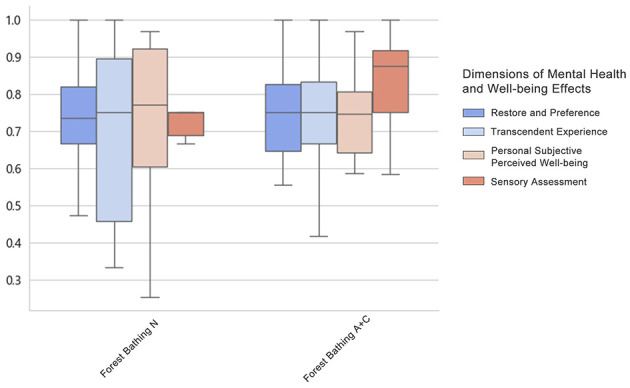
Relationship between forest bathing activities and mental health and wellbeing effects.

The overall mean levels of the four dimensions of the mental health and wellbeing effect produced by FBN were distributed between 0.70 and 0.76, all greater than 0.5. The overall mean levels of the four dimensions of the mental health and wellbeing effect produced by FBC were distributed between 0.73 and 0.84, all greater than 0.5. The mental health and wellbeing effect produced by FBC was higher than that produced by FBN. The overall mean level of the four dimensions of the effect was higher than the mental health and wellbeing effect generated by FBN (Figures 19, 20).

#### 5.4.2 Activities in sensory therapy environment

**Figure 21:**
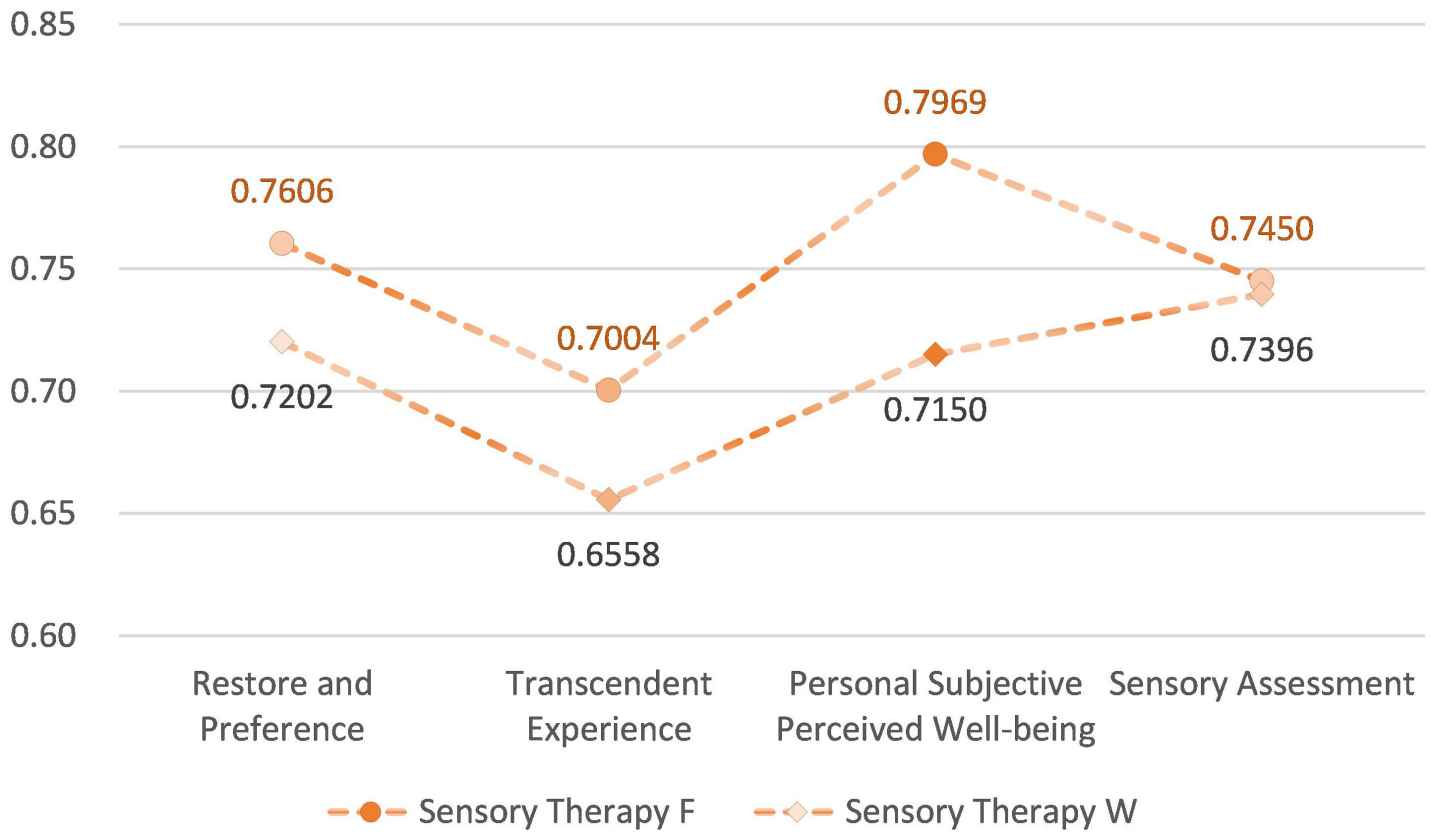
Overall mean distribution of the four dimensions of mental health and wellbeing effects from sensory health activities.

**Figure 22:**
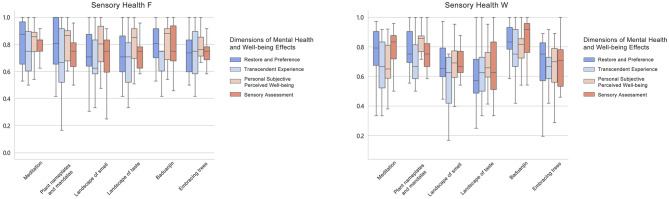
Relationship between sensory health activities and mental health and wellbeing effects.

The overall means of the four dimensions of mental health and wellbeing effects after STF were distributed between 0.70 and 0.80, all greater than 0.5. There was significant variability between the dimensions of Transcendent Experience and Personal Subjective Perceived Wellbeing. The types of recreational activities that produced the highest rankings for Restore and Preference and Personal Subjective Perceived Wellbeing were: meditation, baduanjin, plant nameplates and mandalas. Transcendental Experiences were meditation, embracing trees and baduanjin. Sensory Assessments were meditation, baduanjin, and embracing trees. The overall mean distribution of the four dimensions of mental health and wellbeing effects produced after STW ranged from 0.66 to 0.74, which were all greater than 0.5. There were no significant differences in the effects of the dimensions. The types of recreational activities that produced the highest rankings of Restore and Preference and Transcendent Experience were all: baduanjin, plant nameplates and mandalas, and meditation. Individual subjective perceptions of wellbeing were plant nameplates and mandalas, baduanjin, and landscape of smell. Sensory assessments were baduanjin, meditation, and plant nameplates and mandalas (Figures 21, 22).

## 6 Discussion

In this study, 32 urban healthcare workers entered the GPNP in China to participate in experiments on the physiological and psychological health and personal wellbeing benefits of forest therapy and cultural ecosystem services (CES). The results preliminarily supported the effectiveness of forest therapy and CES in national parks in promoting urban healthcare workers' physiological health benefits, such as improving concentration, neurotransmitter system, immune system, respiratory and circulatory system, enhancing physical fitness, improving sleep, psychological restoration and accessing to wellbeing benefits. This study further identified differences in the health and wellbeing benefits of different recreational activities and types of CES in different environments of national parks. Scientific evidence is provided for the full range of impacts of nature reserves such as national parks on human health, and practical natural solutions for urban healthcare workers and the wider population in terms of health and wellbeing. The most relevant findings of this study are specified below.

### 6.1 Immediate physiological health effects of forest recreation on urban healthcare workers in the GPNP

Results from EEG, BP, and HR indicated that both forest bathing and sensory therapy activities in GPNP may induce varying degrees of relaxation and concentration effects. This exploratory finding further supports the restorative effects of green spaces on people's physical and mental health (Yetkin and Akpinar, 2025). Specifically, from the EEG results, we found that sensory therapy activities by the water exerted a significant relaxing effect, while those in the forest produced significant positive effects on both individuals' relaxation and concentration levels. This is consistent with the findings of our previous study, confirming that compared with visual landscapes alone, the sounds of water and birds can significantly enhance the level of physiological recovery. Among them, hydrodynamic landscapes demonstrate significant physiological health benefits for high-stress populations such as hospital doctors and patients.

Our previous research has demonstrated a positive linear correlation between physiological restorativeness and hydrodynamic landscapes. Regardless of the presence or absence of bird sounds, high hydrodynamic landscapes consistently deliver the highest level of physiological restoration effects for young college students. In the present study, compared with waterfront, sensory therapy activities in forest yielded more significant benefits in enhancing concentration. This observation may be attributed to the fact that, in contrast to the waterfront environment with high hydrodynamic landscapes, the forest environment, characterized by low-to-moderate sound pressure levels, is relatively quieter, and its auditory stimuli primarily originate from bird sounds in the forest, which is more conducive to improving human concentration (Han, 2003).

Furthermore, we also found that forest bathing in natural environments with cultural elements significantly enhances individuals' concentration, and this finding aligns with the observations made during our experiment. Specifically, in the experiment, accompanying staff observed that when participants engaged in forest bathing in the culturally infused environment, they exhibited a state of deep immersion upon viewing the Forestry Heroes Monument, the illustrations in the popular science corridor, and the rich collection of butterfly specimens in the ecological museum. Such immersion experienced under natural relaxation may contribute to the significant improvement in concentration.

The study further identified that sensory therapy activities in forest exerted significant positive effects on both individuals' relaxation and concentration levels. From the perspective of the neurotransmitter system, the forest environments, waterfront environments, and culture-infused natural environments within the national park, as well as the observation and experience activities conducted in these settings, may be more conducive to evoking positive or even excited emotions. During the experiment, the accompanying staff observed that participants exhibited intense affection when viewing a rich variety of plants, or when they looked up, bent down to observe and take photographs. When encountering wild animals such as sika deer, wild boars, takins, and *Chrysolophus pictus*, the participants showed great excitement, even cheering. In the culture-infused natural environment, the participants took selfies and group photos joyfully, and paused to view humanistic landscapes, popular science content, as well as promotional videos that are vivid, rich in content and grand in momentum. The sense of observation, experience and interaction in these processes can stimulate individuals' curiosity and desire for exploration and self-aesthetics, which in turn induces the secretion of dopamine (Tian et al., 2014), and helps to enhance individuals‘ sense of wellbeing and satisfaction.

In addition, water features with hydrodynamics can increase the active atmosphere and vitality of the environment in addition to their morphology, and the sound of running water emitted by them can likewise produce a bright and lively, positive and pleasant feeling (Luo et al., 2023), and medium- to high-hydrodynamic water features in a green background can also suggest a sense of vitality. This is in line with our findings with previous studies. Previous studies have indicated that disturbances in DA and NE are associated with depression and cognition (Wang et al., 2025), and depression can be treated with medications that regulate DA levels (Elhwuegi, 2004). The findings of the present study support that forest bathing and sensory therapy activities in different environments exert a significant positive effect on DA. This finding also prompts us to consider whether, for patients with depression, forest bathing and sensory therapy activities in national parks could potentially be incorporated as “green prescriptions” for adjuvant therapy in the future, in addition to pharmacological treatment. In terms of the respiratory, circulatory, and immune systems, the present study found that both forest bathing and sensory therapy activities in national parks may help alleviate inflammation in the small airways of the human body, thereby promoting the improvement of respiratory health. This may be associated with the vegetation environment in the deep forest, which features high richness and canopy density. Vegetation in this environment absorbs air pollutants such as haze (Guo et al., 2010) and reduces the damage of pollutants to the respiratory tract, thereby alleviating the inflammatory response, maintaining homeostasis of respiratory health, and effectively preventing respiratory diseases (Nowak et al., 2018). Forest bathing and sensory therapy activities in national parks also contribute to immune responses and enhance the ability to regulate immune cell activity. This is consistent with the findings of previous scholars that after forest bathing, the activity of NK cells in human blood was significantly increased, and the amount of anticancer proteins also increased dramatically (Li, 2010). NK cells are able to induce apoptosis of cancer cells (Li et al., 2005). This may be related to the characteristics of the forest itself, which is rich in abundant health benefit factors such as sufficient oxygen, negative ions, and body-benefiting plant essences (Qie et al., 2011). Furthermore, the role of forest bathing in culturally infused environments in enhancing immune system function deserves special attention. This may be attributed to the fact that the forest environments with cultural elements in this area also possess a highly favorable ecological climate (see the environmental data in Tables 2, 3), while the cultural elements serve as an added advantage. In summary, the research findings regarding the respiratory and circulatory systems, immune system, and neurotransmitter system remind us that culture and nature are not mutually exclusive. The integration of cultural elements into a sufficiently high-quality ecological environment may bring additional benefits to humans. This exploratory finding may provide insights for the future selection of wellness microenvironments targeting sub-healthy populations and populations with respiratory diseases.

### 6.2 Psychological health and personal wellbeing benefits of forest recreation and CES in GPNP for urban healthcare workers

The experimental and scale data from this study revealed that the CES values obtained by urban healthcare workers in the GPNP environment and the implementation of forest bathing and sensory health activities in different environments can produce psychological health and personal wellbeing effects. The perceived values of aesthetics, biodiversity, sustaining life, spirituality, recreation, sense of place, and healing were higher. This may be due to the high value characteristics of the perceived object, i.e., the GPNP itself, i.e., the well-preserved natural ecosystems and forest vegetation in Tangjiahe Area of the GPNP, with a very high degree of biodiversity, which contributes to the contact and connection between human beings and nature, and thus helps urban healthcare workers to harvest high levels of aesthetic and biodiversity value perceptions.

Both actual and perceived levels of biodiversity have been shown to be closely related to the health and wellbeing of residents (Marselle et al., 2021). And the process by which respondents gain psychological restorative benefits through perceived biodiversity characteristics of green spaces should be the most intuitive way to benefit (Wang et al., 2021). This experimental area is a national nature reserve with giant pandas, twisted-horned antelope and their habitats as the main objects of protection, and rare wild animals were also encountered with high frequency in the forest bathing experiment in the natural environment of this study, therefore, subjects could perceive richer plant and animal species in the environment, which is more conducive to the proximity to and interaction with nature thus contributing to the higher health and wellbeing benefits. It also facilitated the generation of a sense of sustained life and emotions of awe, humility, and reflection among the urban healthcare workers, which supports that encountering wildlife in natural environments contributes to a transcendental experience for people. National parks are excellent places for healing and recuperation, and forest bathing walks and respiratory function activities as well as sensory recuperation activities in the GPNP can not only stimulate the sense of fun of the participants and obtain the generation of recreational value through multi-sensory stimulation, but also effectively reduce people's mental stress and physical fatigue, and then promote physical and mental health (Deng et al., 2020; Franco et al., 2017), realizing the healing effect of forest recuperation.

This study has made exploratory findings that the most contributing activities to the mental health of urban healthcare workers in different environments were not exactly the same, with Baduanjin, botanical nameplates and mandalas, and meditating on positive thoughts being highly contributing to both types of environments, while the activity of aroma-viewing recreation was more contributing in the waterside environment of a national park, and the activity of embracing a large tree was more contributing in the forested environment of a national park. It is possible that this is influenced by both the activity itself as well as the environmental differences. In the ecological setting of a national park, one's natural affinity for water (Kaplan and Kaplan, 1989) may potentially allow one to experience a deeper level of relaxation and comfort than in the more secluded setting of a forest. Therefore, it is easier to fully mobilize the senses of taste and smell to obtain a richer experience when conducting aroma-scape recreation activities in waterside environments. Compared with waterfront areas, forest environments are quieter and more confined, with lush plant growth and higher oxygen content. This also suggests that forest therapy environments do not have consistent physiological and psychological health effects on people. To date, visual perception has figured prominently in forest landscape perception studies. Several studies have shown that moderately dense, tall, and neat forest landscapes are the most popular (Chiang et al., 2017; Edwards et al., 2012). Tactile sensation and visual viewing may produce different feelings; through tactile sensation, people can feel the texture, temperature, form, and other information of the trees, which helps participants feel the inherent natural power, gain a sense of stability and security, and moreover, helps participants to relax and experience the connection with nature (Pálsdóttir et al., 2014). In addition, this study also found that the natural environment with humanistic atmosphere is higher than the mental health effect produced by forest bathing in natural environment, which induces more autonomous activities. This may be related to the group, social and cultural nature of human beings. Humans are naturally social animals and tend to interact and relate to others. Humanistic natural environments not only allow people to experience the beauty of nature, but also provide more opportunities for people to engage in various activities, such as hiking, photography, and cultural exchanges, which can enhance a sense of belonging and social support, and have a positive effect on mental health.

## 7 Conclusion

In this study, we systematically explored the effects of forest therapy and CES (as a secondary background element) in the GPNP on the physiological and psychological health of urban healthcare workers, and preliminarily supported the effectiveness of nature reserves such as national parks in promoting physiological health improvement, psychological recovery, and the acquisition of wellbeing benefits for urban healthcare workers by conducting comprehensive and systematic measurements of physiological and psychological health and wellbeing benefits indicators.

We have observed that both forest bathing and sensory therapy activities within the GPNP may yield varying degrees of relaxation and concentration benefits. Forest therapy in medium hydrodynamic landscapes may offer significant physiological relaxation benefits for high-stress groups such as urban healthcare workers, while sensory therapy in forest environment may positively enhance concentration levels. Activities such as observation and experiential learning within national parks characterized by pristine ecological environments may be more effective in evoking positive or even exhilarating emotions. This exploratory finding could potentially contribute to the rehabilitation treatment of individuals with depression.

Research findings on respiratory and circulatory systems, immune systems, neurotransmitter systems remind us that culture and nature are not in conflict. Infusing cultural elements into sufficiently good ecological environments may bring greater benefits to humanity. This exploratory discovery could aid future selections of therapeutic microenvironments for sub-health populations and individuals with respiratory diseases.

This study also found that the most contributing activities to the mental health of urban healthcare workers in different environments were not exactly the same, with Baduanjin, plant nameplates and mandalas, and meditation on positive thoughts being highly contributing to both types of environments, while the landscape of smell was more contributing in the waterside environment of a national park, and the activity of embracing trees was more contributing in the forested environment of a national park. Additionally, the mental health benefits derived from natural environments with cultural ambiance surpass those of forest bathing in purely natural settings, which aligns with our findings regarding physiological benefits.

In terms of population selection, this study focuses on urban healthcare workers, a group that often faces high levels of stress and emotional strain during their careers, and provides specific data and insights on the effectiveness of natural environments in relieving stress in people with high occupational stress. Future research could target broader populations, including vulnerable groups, by employing forest bathing as an intervention that leverages various elements of the forest environment to alleviate stress and promote physical and mental health. Additionally, we have conducted comprehensive studies across spring, autumn, and winter seasons to further explore the interactions between temporal variations, environmental conditions, and physiological and psychological restorative effects.

However, due to the complexity of outdoor environments, especially wilderness environments, the lack of analysis of the microenvironment could not be avoided, even though the subject has tried to consider the control of environmental variables. The study also still has limitations in terms of seasonal data collection and systematic assessment of long-term health effects. To address these limitations, future research should consider conducting comparative experiments in different seasons to assess the impact of seasonal changes on health effects, and gradually launch experiments on more sub-healthy and diseased populations in national parks, to more widely realize the formulation of forest green prescriptions and the all-around impacts of nature reserves, such as national parks, on human health to provide more comprehensive and in-depth scientific evidence, and at the same time, to promote the application of forest recreational activities in the global health. Finally, we consider important to promote the application of forest therapy activities in global health policies and nature conservation strategies, and provide practical natural solutions for the health and wellbeing of people in high-stress occupations.

## Data Availability

The data are not publicly available due to restrictions e.g., their containing information that could compromise the privacy of research participants. Requests to access the datasets should be directed to Ping Zhang, pingzhang@sicau.edu.cn.
